# Improving food safety in the informal sector: nine years later

**DOI:** 10.1080/20008686.2019.1579613

**Published:** 2019-03-08

**Authors:** Delia Grace, Morenike Dipeolu, Silvia Alonso

**Affiliations:** a International Livestock Research Institute, Nairobi, Kenya; b College of Veterinary Medicine, Federal University of Agriculture, Abeokuta, Nigeria

**Keywords:** Foodborne disease, intervention, informal sector, Nigeria

## Abstract

**Introduction:** Foodborne disease is a major public health problem in poor countries, but we lack effective, sustainable and scalable approaches that work in the traditional, informal markets where most fresh, risky food is sold. A promising intervention is working with informal sector vendors to provide: a) training and technologies; b) an enabling environment; c) motivation for behaviour change.

**Materials and methods:** We present a long-term follow-up of pilot project in one of the largest abattoirs and meat markets in Nigeria. An evaluation shortly after implementation found the intervention was acceptable, cost-effective and resulted in safer meat.  The follow-up nine years later using mixed methods: qualitative surveys and microbiological tests.

**Results and Discussion:** The policy environment had become disabling, partly as a result of authorities attempts to move butchers to a modern, hygienic but more distant abattoir. This was resisted by the butchers. Authorities revoked the license for Bodija market and stopped providing services. Matters escalated and forceful attempts to remove butchers resulted in deaths followed by riots. Meat safety deteriorated.

**Conclusion:** The case study shows the importance of an enabling environment and need for stakeholder collaboration in attempting to improve food safety in the traditional sector.

## Introduction

Historically, foodborne disease (FBD) has not been seen as a top-ranking public health priority. This changed when the first study on the global burden of FBD study found the health burden was comparable to that of the ‘Big Three’ (malaria, HIV/AIDs or tuberculosis). Unsurprisingly, most of the burden (98%) fell on low and middle income countries (LMICs) but surprising to many, most (97%) of the burden was due to biological hazards [1]. Moreover, the foods most often implicated were the highly nutritious animal source food and fresh vegetables [2,3].

This concern over the health burden of FBD has spurred efforts to improve FBD management. In LMICs, interventions have mainly focused on three nodes of the value chain: production, aggregation steps and households.

- At production or farm level, efforts have focused generally on good agricultural practices and specifically on management of individual hazards such as *Brucella spp*. in dairy cattle or aflatoxins in maize [3].
- Along the value chain, efforts have been targeted to aggregating steps: that is, dairy plants, slaughterhouses and wholesalers. These are both important contamination nodes, and because a majority of food consumed may be aggregated, they are a leverage point where one intervention can benefit many thousands of consumers. Moreover, it is easier to install infrastructure and to inspect products at one large centralized aggregating point than at dozens or hundreds of smaller aggregating points [3].
- At household level, a meta-analysis suggested some success in informing and training those responsible for meal preparation (usually women). However, the sustainability, scalability and practicality of these remains un-clear [4].

The International Livestock Research Institute (ILRI) is a research organisation with the mandate to discover and develop solutions that maximise the contribution of livestock to nutrition, health, livelihoods and the natural environment. ILRI started a dedicated food safety agenda in the early 2000s: this initially focused mainly on farms and farmers, especially dairy farmers [5].

In Kenya, improving productivity of smallholder dairy farmers has been a long-standing objective of ILRI. As the dairy sector took off, ILRI realised market access of farmers was under threat, because most milk was sold through informal sector traders and a broad group of stakeholders was coalescing to oppose this. On the one hand, the public health sector believed all milk should be pasteurised because this has long been considered one of the most effective public health interventions. On the other hand, the formal dairy sector opposed the informal which sold cheaper and more accessible milk, on the grounds of ‘lack of a level playing field’ [6].

In this heated debate, ILRI led an initiative to train and certify dairy vendors. This initiative showed that trained vendors produced acceptably safe milk and helped develop a licensing and certification scheme which legitimised the traders [7]. This secured livelihoods, provided markets for smallholder farmers, and ensured cheap milk was still available to consumers. An economic assessment found benefits of $26 million a year [8]. This success, fuelled interest in leveraging the informal sector for improved health, livelihoods and nutrition. To date, interventions have been carried out or are underway in several countries including India, Cambodia, Kenya, Ethiopia and Burkina Faso.

The approach was used in different value chains, including meat, and while the actual intervention was very context specific, researchers identified three components they considered were essential for success [9]: the so called ‘three-legged stool’ model:

- Training and technologies: informal sector actors needed the tools to deliver safe food. This usually meant training, awareness raising and simple technologies such as disinfectants. Training in business skills was often included.
- Enabling environment: regulatory authorities had to be on board with the intervention and there had to be some mechanism for institutionalisation (such as a locally or nationally recognised certificate) and a means of quality assurance.
- Motivation and incentives. These are essential for behaviour change but very context specific. In one case, certificates protected traders against harassment from authorities, in another, training allowed them to improve their bargaining power with the public sector. It was originally hypothesised that trained traders would be able to charge a premium for safer food but in no project were they able to charge more for food though some may have increased market share.

This triple approach sometimes called ‘Training, Certification and Marketing’ or TCM where ‘training’ refers to the capacity building aspect, ‘certification’ to the enabling environment, and ‘marketing’ to the provision of incentives for behaviour change.

## Materials and methods

This paper reports a study to evaluate long term outcomes and impacts from a TCM food safety intervention in an abattoir and associated wet market, in Ibadan Nigeria. We first describe the study site and its history, then summarise the published results of a TCM intervention [10]. Next, we report on a follow up visit, nine years after the original study, to evaluate long-term impacts and the factors influencing these.

### Bodija abattoir

Ibadan is capital of the Oyo state in southern Nigeria and the third largest city in Nigeria with over 3 million inhabitants. When ILRI started work in the mid 2000s, Bodija slaughterhouse and market was the main centre for livestock slaughter, processing and marketing. Live cattle were purchased from all the states in the northern part of the country as well as from neighbouring countries like Niger and Chad. On average, about 150 to 300 cattle were slaughtered daily and there were around 500 slaughterhouse workers. Assuming an average carcase weight of 150kg, and an average meat consumption of 25 g per capita per day (data from FAOStat), this corresponds to a beef-eating population of 1.7 million supplied by the slaughterhouse and market.

At the time of the intervention, Bodija comprised both an abattoir and stalls for retailing meat. The abattoir was built in 1986 with covered roofs and concrete flooring. Water was sourced from a borehole. Abattoir construction was motivated by the common belief that modernising food systems is necessary to improve food supply and will also improve food safety. However, by the 1990s, there had been considerable deterioration in facilities due to lack of maintenance. Animals were slaughtered and butchered on the floor and hygiene was generally low. The abattoir was under municipal management and officers collect tax and tariffs on each cow amounting USD 1 per animal. Environmental sanitary officers inspected slaughter slabs and the general environment. However, the filthy conditions of the market observed by researchers indicated the challenges sanitary officers faced in carrying out their work. The veterinary department was supposed to check animals before slaughtering and inspect meat after slaughter, but many animals escaped inspection and even when problems were found veterinarians find it difficult to ensure condemned meat is discarded, because a condemned animal often represented the only source of livelihood for the butcher for that day.

**Figure 1.**
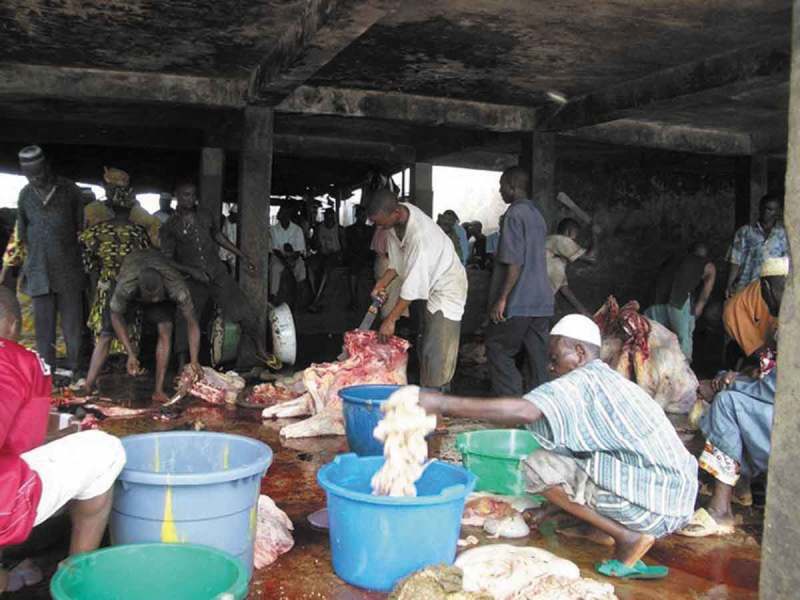
Poor hygienic conditions at slaughterhouse.

Retailers operated from small, covered kiosks in an adjacent part of the market and sold directly to consumers. Hygienic conditions were bad (Figure 1). Meat processing and sale was the main livelihood strategy of butchers; around two thirds of butchers reported it was their only source of income. Both men and women were involved in beef processing and retailing, but among flesh processors (butchers), men predominated. Most workers were self-organised in associations. Butchers’ Associations members included the slaughterers who kill and section the animal but also cattle owners, marketers of live animals, those who brought the cattle to the slaughter slab, leg sellers, skin sellers, head sellers, offal sellers, and meat sellers.

### Intervention for improving meat safety

The ILRI supported intervention study took place between August 2008 and December 2009. The study population comprised butchers in Bodija market. The intervention and short term evaluation are given in detail in Grace et al. [10]. In brief: we identified 16 Associations in the market and then randomly selected 201 meat processors/retailers from the list of members to participate in the TCM intervention. In order to obtain more information on women workers, we subsequently randomly selected an additional 61 women. The intervention was group-based, participatory and interactive. A training workshop was held for Butchers’ Associations representatives in September 2009. The Association representatives were selected by the group and required to pass information and training to other members. The workshop was followed by visits to Butchers’ Associations by researchers in which messages were reinforced and there were discussions around meat hygiene. In terms of the three critical success factors for TCM (the three-legged stool):

- The ‘enabling environment’ aspect was addressed by involving local authorities. However, unlike the Kenya dairy intervention, there was no mechanism for recognition of training or for sustainability in terms of ongoing support.
- The ‘training and technology’ aspect was addressed through simple safety messages and provision of basic equipment to support hygienic handling such as aprons and disinfectants.
- ‘Incentives’ or motivation for behaviour change had two elements. Firstly, butchers were provided with marketing material to draw attention to their training and better practices (banners, tee-shirts, stickers and posters). Secondly, because the intervention was group-mediated, the group was encouraged to put peer-pressure on members to comply.

The cost of the training workshop was $4,414 dollars ($2,528 for materials for improving hygiene, $789 for advertising that drew attention to improved quality and $1,097 for the training).

The intervention was evaluated by comparing knowledge, attitude and practice as well as the microbiological quality of meat, before and after the intervention [10]. Participants significantly improved in knowledge and nearly all reported to have improved their behaviour after the training. When asked what motivated changed behaviour, 77% said it was the training received, 20% said it was because of concern over health and hygiene, 2% said it was because of desire to increase sales, and 2% because of the cleaning inputs received. Participants followed up on their commitment to training other butchers: on average they shared information with another 42 people. After the training, there were highly significantly (p < 0.001) reductions in the proportion of unacceptable samples, across all three parameters of microbiological quality. The reduction was greatest for coliform bacteria (an indicator of faecal contamination).

### Evaluation nine years after the intervention

Nine years later, the project implementation team wished to assess the longer term impacts of the intervention. Visits were made to Bodija Municipal abattoir to investigate the level of compliance to hygiene practices and other intervention measures provided for meat processors and allied workers in the abattoir to ensure safety of meat processed at Bodija Municipal abattoir/meat market. During the visits, leaders of two of the Associations, an official of the Veterinary Department of the Ministry of Agriculture and Rural Development, Oyo State as well as the overall leader of the umbrella-body of all the existing Trade Associations in the abattoir were interviewed. A check-list of questions was prepared for the interviews with the various groups. The questions dealt with a spectrum of issues including the following: operation of the abattoir and market; the type of formal and informal associations that exist; changes since the time of the intervention; current status of the TCM intervention; evolution of the TCM intervention after the end of the project; opinions on drivers of change; and, recommendations. Open questions were used to explore how the situation had evolved in the preceding nine years and to elicit the opinions of interviewees on the factors influencing change.

A study was also conducted on the microbiological quality of meat. Overall, 175 beef samples were collected and examined for specific foodborne bacterial pathogens including *Salmonella enterica, Vibrio cholerae, Shigella* species and enterohaemorrhagic *Escherichia coli* (EHEC) serogroups O157, O26, O45, O91, O103, O111, O121, O128 and O145. The total aerobic count (TAC) and coliform count (CC) were determined as previously described (Mritunjay & Kumar, [11]).

Specific foodborne bacterial pathogens were isolated and identified using conventional microbiological methods including pre-enrichment, selective isolation, biochemical characterization and microscopy (*Salmonella enterica, Vibrio cholerae, Shigella* species and enterohaemorrhagic *Escherichia coli* (EHEC) serogroups O157, O26, O45, O91, O103, O111, O121, O128 and O145). Serological tests were also used in the identification of enterohaemorrhagic *Escherichia coli* (EHEC) serogroups O157, O26, O45, O91, O103, O111, O121, O128 and O145.

Ethical clearance was obtained and participants consented to be interviewed and for samples to be collected and analysed.

## Results: nine years after a food safety intervention

A major change is that the abattoir no longer exists officially at the site. This is because the State Government with private sector partners had developed a new abattoir in Amosun Village, Akinyele. This is called Ibadan Central Abattoir and modern facilities for slaughter and processing of meat were provided in 2014 through public private partnerships. Built on 15 hectares of land with provisions for manual and mechanical slaughtering of cattle, pigs, goats and sheep, this is one of the largest abattoirs in West Africa. The abattoir has stalls for 1000 meat sellers, 170 shops, administrative building, clinic, canteen, cold room, incinerator and a car park that could accommodate 300 vehicles.

In 2014, after the new facility opened, the government revoked the license of Bodija abattoir to operate allegedly owing to unhygienic practices of meat handling by the butchers. Our interviews found that the butchers vehemently resisted the relocation and while some relocated, most of these moved back to Bodija. Although the State Government did not approve the return of the butchers to the Bodija, there was initially no attempt to stop the butchering and retail activities.

When asked why butchers were unwilling to relocate, the respondents said that the new Akinyele abattoir was too far away from the main city where customers seek to buy meat. There were also complaints because the tariff for slaughter increased from 1,000 Naira in Bodija ($2.75 US) to 3,000 Naira ($8.25 US) in the new abattoir. Moreover, those that complied with Government directives and relocated at an early stage complained of poor patronage/sales at the new abattoir, spoilage of unsold meat and loss of capital. There were also rumours that the relocation was politically motivated to benefit the private investor in the new abattoir and that some leaders of butchers’ associations were colluding with authorities and private sector to promote the new abattoir against the interests of their members. On the other hand, several leaders and members of Butchers’ Associations praised the modernity and improved facilities of the new abattoir and market and urged the butchers to relocate. The press was almost uniformly in favour of the new abattoir, often claiming that the old premises were unsanitary and unsafe.

In mid-2018 efforts to relocate the butchers to the new abattoir stepped up. The government ordered all butchers from eleven local government areas in Ibadan, including Bodija market, to relocate to the new abattoir. The butchers argued that the authorities did not have the power to remove them forcibly. In June 2018, newspapers reported that five people were killed at Bodija abattoir market when a security team detailed to enforce movement of butchers to the new center clashed with crowds. In retaliation, butchers attacked the local police station and burned it down. Currently, in late 2018 there has been no decisive resolution of the issue, with further clashes occurring in October 2018, several cases in the courts, and a very contentious arena involving political parties, a non-governmental organisation supporting workers’ rights, Butchers’ Associations and authorities.

When asked to suggest improvements, respondents were overall not satisfied with the new abattoir and suggested that Government should provide more modern abattoirs at different locations within the Ibadan metropolis, thus meeting both objectives of improved hygiene and ready access to consumers.

During our evaluation, recall and impressions of the TCM were also assessed. Interestingly, butchers still remembered the intervention programme and the meat safety measures introduced to the processors. They could also recall essential hygiene practices taught during the intervention workshop. However, they reported that they rarely put these good measures to practice in their daily operations. The materials given by the project to support food safety were also appreciated (rubber boots, aprons, carts and disinfectants). These had been intended by the project as start-up (pump-priming) material which the butchers would replace as needed. The butchers reported that they had used the materials to improve safety of meat in the meat market. However, none of the butchers reported that they continued to buy and replace the materials after the exhaustion of those distributed during the intervention programme. Moreover, some respondents reported that materials distributed were stolen by unknown persons from the store where these materials were kept.

Comparing the indicators of microbiological quality, it can be seen there is a marked deterioration from the post-intervention results and the microbiological quality in 2018 is worse than even before the intervention (see Table 1).

**Table 1.** Meat samples complying with standards before an intervention, immediately after and nine years later.

	Coliforms unacceptable (%)	TAC unacceptable (%)
Bodia before intervention	65.5	97.5
Bodija after intervention	23.5	78.5
Bodija 9 years later	92	100

TAC: total aerobic count (a measure of contamination with aerobic bacteria)

## Discussion

The intervention was a small pilot, with a limited budget. Although the findings were shared with local stakeholders, there was no provision to continue support after the end of the project. As such, we expected the short-term benefits to gradually fade out as there was no change to the institutional structure and no means to refresh the lessons learned or to train new butchers. Meanwhile, the ‘three-legged stool’ approach to tackling food safety in informal markets was increasingly refined and a theory of change developed for its generic application to informal markets [9]. The TCM approach became the central pillar through which the CGIAR aimed to improve food safety in informal markets in the CGIAR Research Program Agriculture for Nutrition and Health. As such, there was interest in finding out what happened to earlier attempts to deploy TCM in informal markets and we conducted a follow up study in 2018 to see what, if anything, remained of the intervention carried out nine years previously.

To our knowledge, the ILRI supported TCM intervention was the first and only initiative to provide training, equipment and motivation necessary to produce safer meat to butchers in Bodija market. The short term evaluation showed it was successful in improving knowledge, practices and microbiological quality of meat. However, without any fundamental change to institutions or mechanisms for re-enforcing practices, the intervention was not sustainable. This supports the frequent observation that one-off training and awareness raising will not be sufficient to attain long term change in practice. The use of appropriate hygiene technologies also has not continued. This is not surprising because, in general, group management of common business resources by poor value chain actors presents considerable challenges of co-operation, co-ordination and incentives for mismanagement [12].

However, it was encouraging to find that even after nine years this relatively simple and low cost intervention was still positively remembered by butchers. It is often said in food safety that many people are well-intentioned but ill-informed, and these people usually respond positively to trustworthy information on how to make food safer for themselves and their customers. Moreover, informal sector actors have been remarkably neglected in agri-food chain interventions and the rarity of support received probably made the TCM intervention more memorable.

The case study also provides an example of how attempts to upgrade value chains can be problematic if they do not take into account the context and the complexities of governance. The modern abattoir had objectively better facilities, but the location was less convenient and the costs for the butchers higher. It was not apparent that any market survey had been carried out to establish demand. There are many other examples in developing countries where modernisation of infrastructure resulted in facilities that were less acceptable to traders and customers. For example, a well-documented case from Lusaka examined how street vendors were moved into new and hygienic premises. However, most returned to their former positions as the improved market was less accessible to customers and entailed more transaction costs for traders, even though the environmental conditions were better [13].

A public-private partnership (PPP) is a contract between government and a private company under which the private company finances, builds, and operates some element of a public service and in turn gets paid over a number of years, through charges paid by users, by payments from the public authority, or a combination of both. They are increasingly promoted in LMIC food systems as a way to remove burdens from over-stretched public resources, access new investments and harness the power of the private sector. However, in low governance and low trust contexts they can under-deliver services, and introduce new opportunities for fraud, collusion, and corruption [13]. Even when there is no evidence of fraud or collusion, the perception of it can erode trust.

The microbiological quality of meat sold in Bodija market is worse in 2018 than before the intervention. The many changes and lengthy time interval between the three microbiological surveys makes it difficult to definitively identify contributing factors, but it seems likely that the highly contentious process of forced relocation and the removal of government services from the abattoir from 2014 onwards led to a deterioration in food safety and increased risk to the public.

## Conclusion

This study supports the hypothesis that while training and provision of simple technologies can improve knowledge, practice and microbiological quality in the short-term, for sustainable improvement additional support is needed. We suggest this includes institutional changes to ensure capacity-building incentives are not short-lived but continuous and there is provision for repeated capacity building. Group ownership of hygiene materials that are intended to be used by individuals may be difficult to support over time. We also present evidence that attempts by authorities to upgrade food infrastructure may have unwanted consequences if the new arrangements do not meet the felt needs of traders and consumers and if there is suspicion of conflict of interests. Finally, given the overall lack of long term impacts it is essential to conduct long term evaluation of food safety interventions: pilots never fail, and pilots never scale.
